# 

**Published:** 1893-12

**Authors:** 


					﻿CONTENTS*
I’AGK.
Physical Culture, its Abuse in Foot-ball.
Robert Jx’ewman, M. D.............251
Dyspepsia. Hugh Slevin, M. D........................... 257
Miscellaneous Paragraphs..........;...................  259
House and Home. Conducted by Lillian White............. 262
Table of Contents Volume XL............................ ifi
Editorial—Hub Me...............................  ■ 265
Literary....................  ..........................265
General Notes...........I............................   269
				

